# Comorbidity in adult patients with autistic spectrum disorder.

**DOI:** 10.1192/j.eurpsy.2026.11076

**Published:** 2026-07-31

**Authors:** S. Sorokin, G. Popova, K. Kostenkova

**Affiliations:** 1 Department for the Study of Endogenous Mental Disorders and Affective States; 2 Department of Early Childhood Psychiatry, Mental health research center; 3 Psychiatric Hospital no. 1 Named after N.A. Alexeev, Moscow, Russian Federation

## Abstract

**Introduction:**

Autism spectrum disorders (ASD) are one of the important problems of modern psychiatry. Despite the fact that the diagnosis of ASD in children is gaining momentum and is actively studied, in adults this diagnosis is practically not made due to the low coverage of this problem and insufficient awareness of psychiatrists. Studies show that most people with ASD are diagnosed with at least one or more comorbid disorders, which complicates its diagnosis and modifies the course.

**Objectives:**

To assess the presence of comorbid disorders in adults with ASD diagnosed in early childhood.

**Methods:**

The study included 58 patients (49 men and 9 women; mean age 20.79 years) with a confirmed diagnosis of ASD who either had been followed in our clinic since childhood and presented for repeat evaluation/treatment, or were first-time referrals who provided pediatric medical records documenting ASD from childhood.

**Results:**

Based on the analysis, the initial symptoms (persistent deficits in social communication and interaction, and restricted, repetitive patterns of behavior) persisted in all patients into adulthood with varying degrees of severity. Improvement in socialization and adaptive functioning was observed in 25.8% of cases—predominantly among individuals with higher intellectual functioning and mild intellectual disability. Among patients with moderate (5.1%) and severe (10.3%) intellectual disability, core symptomatology generally remained at a similar level; however, adolescence was frequently complicated by self- and other-directed aggression and marked behavioral disturbances that required medical treatment and prompted clinical visits. Adults with higher intellectual functioning and without comorbid conditions rarely received psychopharmacotherapy. Most adult presentations of individuals with ASD were for the treatment of comorbid psychiatric disorders. The most frequent diagnoses were obsessive–compulsive disorder (22.4%), depressive disorder (17.2%), and anxiety (15.5%), which is consistent with international data. In addition, ASD in adulthood often comes to psychiatric attention due to the emergence of schizophrenia spectrum disorders; in our sample, such disorders were identified in 10.3% of patients, whereas bipolar disorder was recorded in 3.4%.

**Conclusions:**

Increased awareness of comorbidities in individuals with ASD is important to improve clinical assessment, identification and provision of quality care.

**Disclosure of Interest:**

None Declared

